# Retrospective Study of Multimodality Imaging Features of Chondroblastoma

**DOI:** 10.1007/s43465-024-01214-3

**Published:** 2024-07-03

**Authors:** Aashna Karbhari, Antariksh Vijan, Amit Kumar Janu, Ashish Gulia, Suyash Kulkarni, Nitin Shetty, Kunal Gala, Poonam Panjwani

**Affiliations:** 1 Radiodiagnosis and Imaging, Tata Memorial Hospital, Homi Bhabha National Institute (HBNI), Mumbai, India; 2 Department of Radiology, Advanced Centre for Treatment, Research and Education in Cancer and Homi Bhabha National Institute (HBNI), Kharghar, Navi Mumbai, India; 3 Department of Orthopaedic Oncology, Tata Memorial Hospital, Homi Bhabha National Institute (HBNI), Mumbai, India; 4 Department of Interventional Radiology, Tata Memorial Hospital, Homi Bhabha National Institute (HBNI), Mumbai, India; 5 Department of Pathology, Tata Memorial Hospital, Homi Bhabha National Institute (HBNI), Mumbai, India

**Keywords:** Chondroblastoma, Magnetic Resonance Imaging, Bone Tumors, X-Ray

## Abstract

**Purpose:**

To evaluate the multimodality imaging features of chondroblastoma.

**Materials and Methods:**

Retrospective analysis of imaging features of 52 cases of histopathologically proven chondroblastoma from 2010 to 2022 was performed. Radiographs were evaluated for lesion site, location, morphology, margins, matrix mineralization, cortical breach, periosteal reaction, eccentricity, and subarticular extension. Appearance on T1, T2 weighted and post-contrast T1 was evaluated on MRI, with analysis of peritumoral edema and joint effusion.

**Results:**

Mean patient age was 18 years (10–57 years) with male preponderance (*M* = 39; *F* = 13). 75% (*n* = 39) cases involved an unfused skeleton and 25% (*n* = 13) affected a mature skeleton. Appendicular skeleton was involved in 88.5% (*n* = 46) cases and axial skeleton was involved in 11.5% (*n* = 6) cases with all cases involving epiphysis/epiphyseal equivalent. Radiographically, all cases were well-defined geographic osteolytic lesions with a narrow zone of transition, thin sclerotic rim and lobulated [56% (*n* = 29)] or smooth [44% (*n* = 23)] margins. Matrix calcification appreciable in 62% (*n* = 32) cases was ‘fluffy/smudgy’. Chondroblastoma appeared isointense (83%, *n* = 43) on T1 MRI with characteristically low signal and hyperintense foci within (67%, *n* = 35) on T2-weighted images and post-contrast enhancement [heterogeneous lobular (88%, *n* = 46) or septal pattern (12%, *n* = 6)] with all barring three lesions showing perilesional edema. None of the cases of chondroblastoma in our study developed metastasis till last follow-up (mean: 71 months).

**Conclusion:**

Chondroblastoma has distinctive imaging appearance and is often unlike majority other cartilaginous benign lesions due to characteristic low T2 signal on MRI and associated exuberant perilesional edema.

## Introduction

Chondroblastoma (CB) is an aggressive-appearing bone tumor of chondroid origin accounting for approximately 1% of all benign bone neoplasms [1]. Males are more commonly affected (M:F—2:1) with 90% of the cases being encountered in the second and third decades of life [2, 3]. They predominantly involve the epiphysis and/or apophysis of long bones; however, involvement of more atypical sites has been observed with increasing age [4].

Earlier regarded as an epiphyseal chondromatous giant cell tumor by Ernest Codman in 1931, it was reinterpreted as a separate entity by Jaffe and Lichtenstein in 1942 due to its distinct microscopic features [5]. Pathognomonic histopathologic features include ‘chicken wire calcification’, ‘coffee bean’ cells, and pools of hyaline cartilage with few scattered multinucleated giant cells. K36M mutation in H3F3B or H3F3A genes and positivity on immunohistochemical stains like DOG1 and SOX9 have been described in CB [2]. These aid in its distinction from giant cell tumor which is a close differential, on pathology as well as on imaging.

Clinically, it presents with local site pain and/or mass with decreased range of motion due to the inflammatory changes incited in the surrounding tissues [5–7]. Traditionally treated by surgical resection, the increased risk of injury to the articular surface or adjacent open growth plates led to adoption of relatively safer percutaneous ablative methods [8].

Despite the near characteristic location and radiographic features of CB, its non-specific and varying appearance in some cases and exuberant edema often lead diagnosticians astray. Our study aimed to elucidate the characteristic imaging findings that can help in unequivocal identification of CB, obviating unnecessary sampling and guiding appropriate management.

## Materials and Methods

### Patient Selection

Retrospective analysis of 52 histologically confirmed cases of chondroblastoma was performed over a 12-year period (from January 2010 to May 2022) at a tertiary cancer care center. Patients with pre-treatment radiographs and MRIs available on the institutional PACS (picture archiving and communication system) or those with optimal DICOM (digital imaging and communications in medicine) images available on institutional PACS were included.

### Imaging Acquisition

Standard 2-view radiographs were evaluated for all cases. CT studies were acquired on a 16-slice MDCT (Siemens Emotion 16 2010 / GE Lightspeed16) scanner and assessed in sharp kernel (bone algorithm) and soft tissue reconstructions. Multiparametric MR imaging was obtained on 1.5 T (GE Healthcare, Milwaukee, USA), 1.5 T (Philips Medical Systems, Eindhoven, the Netherlands), or 3 T (GE Healthcare, Milwaukee, USA) MRI machines at our institute with T1 weighted, T2 weighted, STIR, and T1 post-contrast sequences being available for all cases. In addition, gradient echo (GRE), diffusion-weighted (DWI), and dynamic contrast enhancement (DCE) were assessed when available. PET–CT using 18F-FDG/ fluoride radiotracer was performed at our institute on a Phillips Gemini TF 16 slice scanner.

### Image and Statistical Analysis

After due ethics committee clearance, multimodality images were reviewed on Centricity PACS RA1000 (GE Healthcare, Barrington, IL).

Radiographic evaluation of the tumor included general morphological parameters such as site (appendicular or axial skeleton and bone involved), location (epiphysis/apophysis, epi-metaphysis, epi-meta-diaphysis), margins (classified using the Lodwick–Madewell classification as type 1—geographic 1A: thin, sclerotic margin. 1B: distinct, well-marginated border, without sclerotic rim. 1C: indistinct border; type 2—moth-eaten; type 3—permeative), matrix mineralization (present/absent/type), cortical breach, periosteal reaction, eccentricity, and subchondral extension. CT studies were evaluated for matrix mineralization (present/absent/type), cortical breach, and periosteal reaction.

MRI studies were assessed for signal intensity alterations on T2WI and T1W (classified visually as low, intermediate and high), and post-contrast enhancement (classified as lobular and septal). Advanced MRI sequences assessed included: DWI (diffusion restriction present or absent), GRE (for foci of blooming suggesting calcific matrix), and dynamic contrast enhancement (DCE) curve where available. In addition, presence of peritumoral edema and joint effusion were also studied. The edema was graded visually as mild, moderate, and severe/exuberant, representing edematous change equal to or less than the lesion diameter, less than twice the lesion diameter, and more than twice the lesion diameter, respectively.

Data were analyzed on MS Excel using frequency and percentage for categorical variables.

## Results

Sixty consecutive cases of chondroblastoma were identified from the bone and soft tissue disease management group database from January 2010 to May 2022, of which eight cases were excluded due to non-availability of pre-treatment imaging on institutional PACS. Fifty-two cases of histopathologically proven chondroblastomas were evaluated, of which 75% cases (*n* = 39) occurred in unfused skeletons with only 25% (*n* = 13) affecting a mature skeleton, with mean age of 18 years (ranging from 10 to 57 years), and a male:female ratio of 3:1 (M = 39, F = 13).

Size for the cases in our series has been studied with 13 cases showing size < 3 cm, 37 cases showing size between 3 and 5 cm, and 2 cases showing size > 5 cm. Majority of the lesions (88.5%; *n* = 46) affected the appendicular skeleton, most commonly involving the proximal humerus (*n* = 16), followed by the femur (*n* = 13) and tibia (*n* = 12). Other less frequently involved appendicular skeletal sites included the scapula, lunate, and calcaneum. Axial skeleton involvement (11.5%, *n* = 6) was limited to the skull base (*n* = 1), temporal bone (*n* = 2), and acetabulum (*n* = 3) in our study.

Some degree of epiphyseal or epiphyseal equivalent involvement was present in all cases affecting the appendicular skeleton (*n* = 46), with 39% cases (*n* = 18) showing exclusive involvement of epiphysis/epiphyseal equivalent. Metaphyseal extension was present in 56% (*n* = 26) cases, with epi-meta-diaphyseal extension in the remaining 4% (*n* = 2). All lesions were confined to a single bone.

**Fig. 1:**
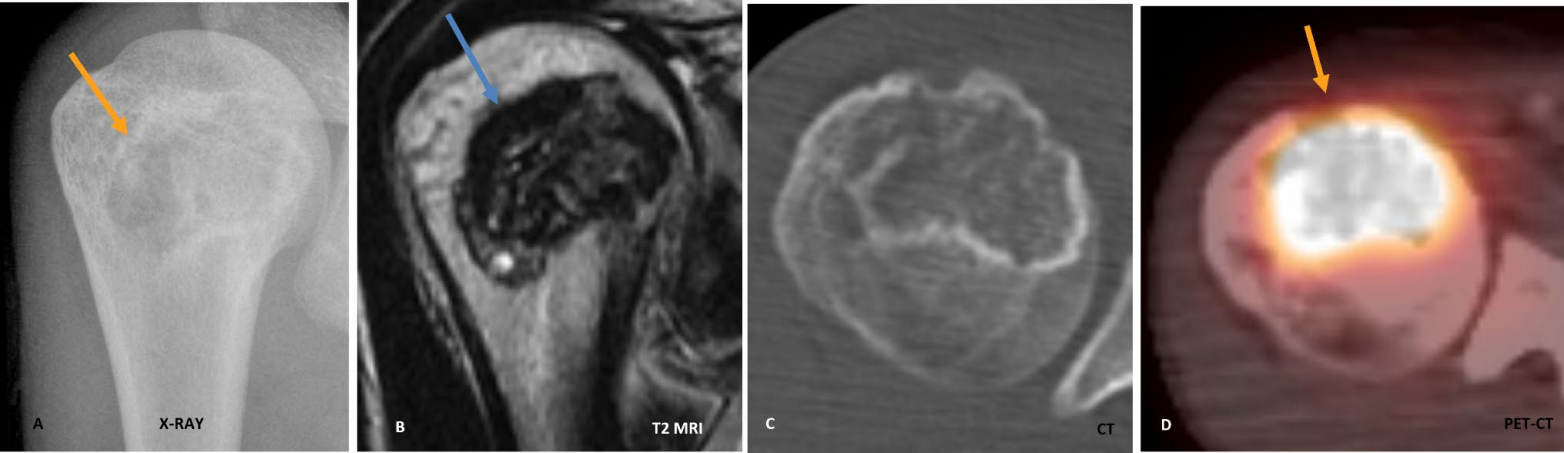
Typical imaging features of chondroblastoma on radiograph, MRI, CT, and PET–CT. **A** Radiograph demonstrates an osteolytic epi-metaphyseal lesion with narrow zone of transition, sclerotic rim, and internal calcification. **B** T2W coronal image through the humeral head shows a T2 hypointense lesion with occasional T2 hyperintense foci. **C** Axial CT image in bone window demonstrates sclerotic rim and internal chondroid type calcification. **D** Fused transverse PET–CT image shows intense FDG-avidity within the lesion

All 52 cases in our study presented radiographically as well-defined geographic osteolytic lesions with a narrow zone of transition and a thin sclerotic rim (Lodwick–Madewell 1A) (Fig. 1). 56% of the lesions (*n* = 29) in our study showed lobulated margins, with the rest 44% of the lesions (*n* = 23) showing smooth margins. While internal calcification has been described as a characteristic feature of CBs, these may be difficult to detect on radiographs alone. About 62% (*n* = 32) cases from our study group showed ‘fluffy/smudgy’ type of matrix calcification, and CT or GRE MRI images served as useful adjuncts in their confident identification.

**Fig. 2:**
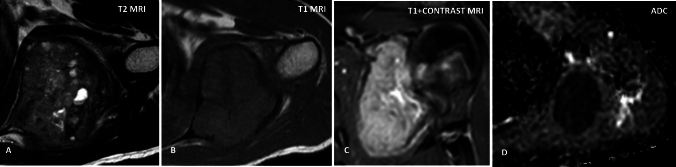
Typical MRI features of chondroblastoma. **A** Axial T2W image demonstrates T2 hypointensity with few interspersed T2 hyperintense foci. **B** Axial T1W image shows a T1 intermediate signal intensity lesion with T1 hypointense rim. Coronal T1 + C (**C**) depicts post-contrast enhancement. In apparent diffusion coefficient map (**D**), the lesion shows significant restricted diffusion (seen as dark signal)

MRI features (Fig. 2) consisted of a predominantly T1 isointense (*n* = 43) lesion with heterogeneous T2 signal intensity. Most of the lesions (67%, *n* = 35) showed the characteristic low T2 signal intensity with T2 hyperintense foci within. GRE images were available for 22 cases, of which all of them demonstrated at least few tiny foci of blooming. All 52 cases showed some degree of post-contrast enhancement, with 88% (*n* = 46) cases showing heterogeneous lobular enhancement and septal pattern being observed in the remaining 12% (*n* = 6).

**Fig. 3:**
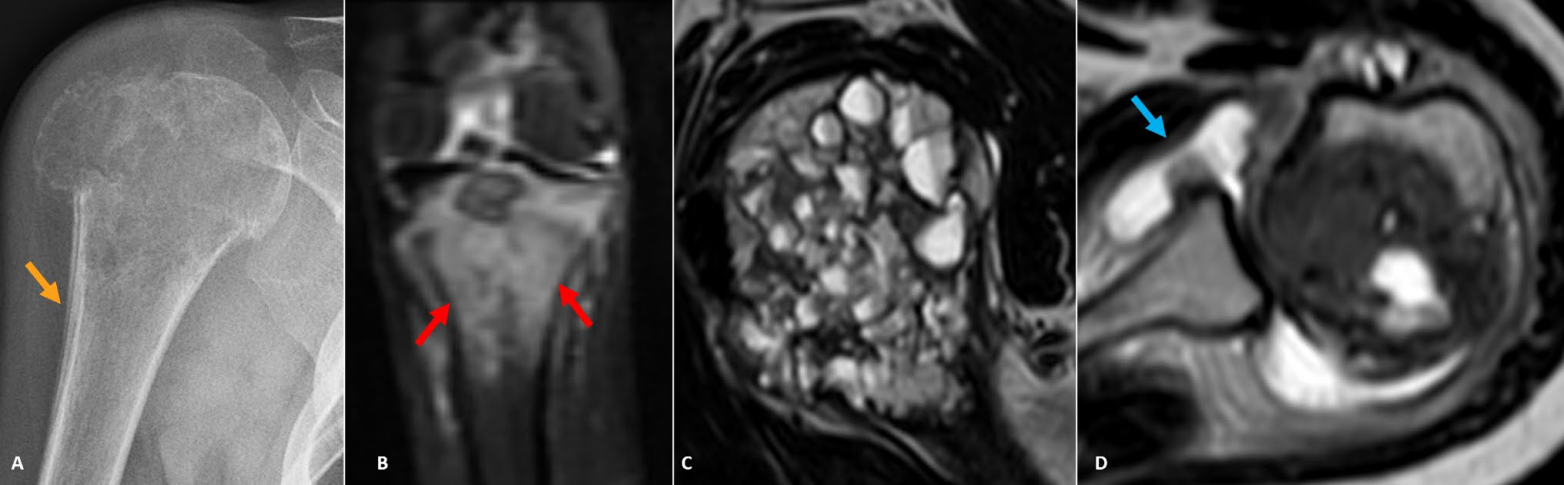
Aggressive imaging features in chondroblastoma. **A** Cortical breach, periostitis, pathologic fracture—radiograph of the right humerus with greater tuberosity chondroblastoma demonstrating cortical breech and linear periosteal reaction (arrow) along humeral meta-diaphysis. Also note minimally displaced pathological fracture. **B** Exuberant edema—coronal STIR MRI image in a proximal tibial chondroblastoma demonstrating exuberant marrow edema (red arrows) disproportionate to lesion size. **C** Fluid–fluid levels—sagittal T2W MRI image showing innumerable fluid–fluid levels in a humeral chondroblastoma. **D** Joint effusion—T2 intermediate to hypointense humeral lesion with articular extension and moderate joint effusion (arrow)

All barring three lesions showed perilesional edema of some degree, with 50% (*n* = 26) showing mild, 36% (*n* = 19) showing moderate, and 8% (*n* = 4) showing severe or exuberant edema. STIR images best demonstrated the marrow and soft tissue edema, along with presence of joint effusion in 50% cases (*n* = 26). We did not find perilesional edema in three cases involving the axial skeleton (viz. temporal bone and skull base). Among the other features that lent CB an aggressive appearance on MRI (Fig. 3) was the presence of periosteal reaction (*n* = 8, ~ 15%). Cortical breach was seen radiographically in about 45% cases (*n* = 23), appreciated better on CT much alike the lesion matrix. None of the cases of chondroblastoma in our study developed metastasis till last follow-up (mean follow up duration = 71 months).

## Discussion

This retrospective review of multimodality imaging in chondroblastomas reiterates its ‘unlike-cartilaginous lesion’ imaging appearance. Our study cohort suggesting male (3:1) preponderance with mean patient age being 18 years (range: 10–57 years) was in line with existing literature [1–3]. As described in multiple previous studies, 75% of our cases were observed in immature skeletons with 46 cases involving long bones showing a striking epi/apophyseal affinity (*n* = 46). Most common site in our study (proximal humerus) was similar to findings of various previous studies [1–3, 9, 10]. Atypical distribution was frequent in older patients, with patients older than 30 years of age showing involvement of scapula (*n* = 1), lunate (*n* = 1), acetabulum (*n* = 1), skull base (*n* = 1), and temporal bone (*n* = 2). This is similar to findings of Bomhauer et al. where atypical short tubular and flat bone involvement [4], higher metaphyseal involvement, and relatively more aggressive behavior in terms of rates of local recurrence and metastases were observed with increasing age. About 47% (*n* = 22) of our appendicular cases were found to have subarticular extension which conventionally has been cited as a classic feature of giant cell tumors (GCT). However, more than half of these cases were non-eccentric, a feature that may aid in morphologic distinction from GCT.

Type of margin and cortical breach were better visualized on CT which was similar to the observations of Weatherall et al. The presence of a characteristic sclerotic rim can help in distinguishing CB from clear cell chondrosarcomas which tend to permeate the outer cortex [11] and GCT which usually lack a sclerotic rim apart from their propensity to afflict older patients and lack of perilesional edema incitement.

Dia-metaphyseal solid or layering periostitis away from the lesion described by Brower et al. [12] in 47% of their cases (101/214) was observed in ~ 15% cases (*n* = 8) in our study. Periosteal reaction, when present, was thick and solid which can hint toward the benignity of this aggressive appearing entity (Fig. 3).

On MRI, this ‘dark horse’ of cartilaginous lesions demonstrated a predominantly low T2WI signal intensity with high SI foci within, in 66% of our cases (*n* = 34). This is in line with the findings of Weatherall et al. who found this characteristic appearance in 16 of their 22 cases (73%) [7] and Jee et al. who observed it in 10 of their 22 cases (45%) [13]. They further found that higher percentage of high SI foci within a lesion corresponded with higher percentage of lakes of hyaline cartilage and/or hemorrhage on histology. Such correlation was not possible in our study as curettage specimens were available in only 20 cases and only representative tissue was sent for histologic evaluation in 32 cases that were treated by radiofrequency (*n* = 31) or cryoablation (*n* = 1). Combination of highly cellular chondroid matrix and calcification has been postulated to cause the decrease in T2 relaxation time [7, 14, 15].

Touted to be a cardinal feature associated with CB, perilesional edema was found in 94% (*n* = 49) cases with moderate to severe (equal to more than the lesion diameter) edema in 44% cases (*n* = 23), often seen extending to adjacent muscles and inciting joint effusion in 50% cases (*n* = 26). J.W et al. encountered joint effusion in 75% of their cases, majority due to direct joint invasion by the lesion with few cases of effusion in the absence of direct joint involvement [14].

Perilesional alterations appearing as subtly increased intramedullary density on radiograph, hypointensity on T1W, and hyperintensity on STIR/T2W MRI images, with radiopharmaceutical uptake on metabolic imaging associated with CB, play a part in its sinister appearance. Locally acting enzymes released by blastoma cells and bone morphogenic proteins (BMPs) have often been held culprit for the peritumoral edema. Weatherall et al. felt this characteristic ancillary finding could not be explained by Frost’s ‘regional acceleratory phenomenon’ or Bower et al.’s ‘stress phenomenon’ alone, though it could be an analog of the ‘flare phenomenon’ described for osteoblastomas by Crim et al. [16, 17].

Another feature lending it an aggressive appearance included associated pathological fractures (*n* = 2); however, they are likely to be exceptions than the rule [6, 18]. Secondary, aneurysmal bone cyst causing fluid–fluid levels have been seen in up to 15% cases of CB; however, we encountered only two cases (acetabulum, femur) showing such an appearance in our cohort.

Like Jee et al. we observed enhancement in all cases, demonstrating either heterogeneous lobular or septal pattern [13]. Dynamic enhancement curves were available for two cases and were of type 2 (plateau), further pointing toward benign etiology (Fig. 1). 50% of the cases showed restricted diffusion (Fig. 2) which was patchy more often than not.

Post-treatment MRI showed increased lesion heterogeneity on T2 and T1 images and continued to show persistent minimal to mild enhancement which was stable or decreased as compared to pre-treatment scan. There was near-complete resolution of perilesional edema on MRI and no radionuclide uptake in the lesion seen on PET/CT which served as the predominant marker for complete tumor ablation (Fig. 2).

We acknowledge the potential bias in the study due to its retrospective nature. Pathologic correlation for specific imaging findings was limited as curettage was performed in only a few cases due to evolving management strategies for benign bone tumors. Finally, there might have been variations in image acquisitions across different scanners; however; non-standardized imaging protocols can perhaps be viewed as a plus, since it suggests that results may be generalized and will likely be less dependent on protocol variations between centers/scanners.

In conclusion, chondroblastoma has distinctive imaging appearance and is often unlike majority other cartilaginous benign lesions due to characteristic low T2 signal on MRI and associated exuberant perilesional edema (Table 1).

**Table 1:** Results

Demographic and general features	
1. Age	Mean age = 18 years Age range = 10 to 57 years
2. Sex	Male:female = 3:1
3. Skeleton fusion	Unfused skeleton = 75% (*n* = 39) Mature skeleton = 25% (*n* = 13)
4. Site	Appendicular skeleton = 88.5% (*n* = 46) Axial skeleton = 11.5% (*n* = 6)
5. Distribution in the appendicular skeleton with respect to physis	Exclusively epi-/apophyseal = 39% (*n* = 18) Epi-metaphyseal = 56% (*n* = 26) Epi-meta-diaphyseal = 4% (*n* = 2)
Radiographic features	
1. Morphology	Osteolytic lesion = 100% (*n* = 52)
2. Margin	Geographic lesion with thin sclerotic rim—Lodwick–Madewell 1A (*n* = 52) Lobulated = 56% (n = 29) Smooth = 44% (*n* = 23)
3. Matrix calcification	‘Smudgy’ calcification present = 62% (*n* = 32) ImPerceptible = 38% (*n* = 20)
4. Eccentricity	Non-eccentric = 57% (*n* = 30) EcCentric = 43% (*n* = 22)
5. Subchondral extension	Present = 43% (*n* = 22) Absent = 57% (*n* = 30)
MRI features	
1. T2WI signal intensity	‘Dark’: Predominantly low signal intensity with hyperintense foci = 66% (*n* = 34) Intermediate = 30% (*n* = 16) High signal intensity = 4% (*n* = 2)
2. T1WI signal intensity	Isointense = 82% (*n* = 43) Heterogeneous = 18% (*n* = 9)
3. Post-contrast enhancement	Heterogeneous lobular = 88% (*n* = 46) Septal = 12% (*n* = 6)
4. Peritumoral edema	Mild = 50% (*n* = 26) Moderate = 36% (*n* = 19) Severe/exuberant = 8% (*n* = 4)
5. Joint effusion	Present = 50% (*n* = 26) Absent = 50% (*n* = 26)
Aggressive features	
1. Periosteal reaction	Present = 15% (*n* = 8) Absent = 85% (*n* = 44)
2. Pathological fracture	Present = 4% (*n* = 2) Absent = 96% (*n* = 50)
PET-CT	
1. SUVmax pre-treatment	FDG avidity, mean 7.2
2. SUVmax post-treatment	Non-avid
Treatment-related and clinical factors	
1. Treatment employed	Radiofrequency ablation = 29 Curettage and grafting = 21 Cryoablation = 1 Conservative = 1
2. Metastases	Absent
